# Supplementary material for: Model Simulation and Rheological Research on Crosslinking Behavior of Polyethylene Resin
Source: Gels. 2023 Dec 31;10(1):35. doi: 10.3390/gels10010035 (PMC10815699; doi:10.3390/gels10010035)
Supplement: Supplementary file 1 [file gels-10-00035-s001.zip › gels-2764856-supplementary.pdf]

Model Simulation and Rheological Research on Crosslinking Behavior of Polyethylene Resin

Xuelian Chen [1,2,*] and Qigu Huang [1,*]

1 State Key Laboratory of Chemical Resource Engineering, MOE Key Laboratory of Carbon Fibers and Functional Polymers, The College of Material Science and Engineering, Beijing University of Chemical Technology, Beijing 100029, China
2 Shenhua (Beijing) New Materials Technology Co., Ltd., CHN Energy Group, Beijing 102211, China
* Correspondence: xuelianchen_nice@163.com (X.C.); huangqg@mail.buct.edu.cn (Q.H.)

S1 the "S" model for crosslinked polyethylene (XLPE)

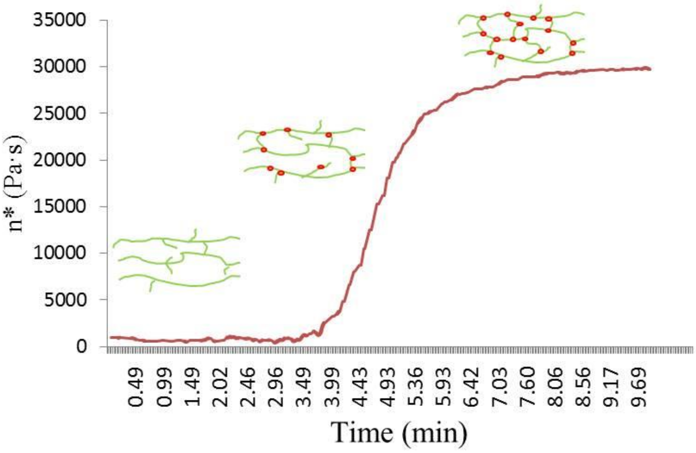

Figure S1. Crosslinking degree of XLPE as "S" model on complex viscosity.

The S-type model refers to the curve that the composite viscosity or energy storage modulus of crosslinked polyethylene will increase with the increase of the amount of crosslinking agent or the extension of the crosslinking time. The S-type model is proposed to predict the change of crosslinking degree value according to the change of the amount of crosslinking agent or the change of crosslinking time through mathematical empirical model.The figure below shows the relationship between processing time and the degree of crosslinking or viscosity of the resin melt. In this work, the mathematical formula was obtained based on the crosslinking mechanism of "S" model XLPE. Therefore, the mathematical formula was called as "S" model.

While the parameters of the S model do not hold any physical significance, they are undoubtedly impacted by the crosslinking process. Therefore, in order to analyze the mechanical process of crosslinking more effectively, we are integrating the Ding model. This model is commonly utilized to investigate the kinetics of rubber vulcanization, and here, we are applying it to examine the impact of different resin structures on crosslinking mechanics.

S2 Effects of loss modulus (G'') by the peroxide mixture content for different types of XLPE resins

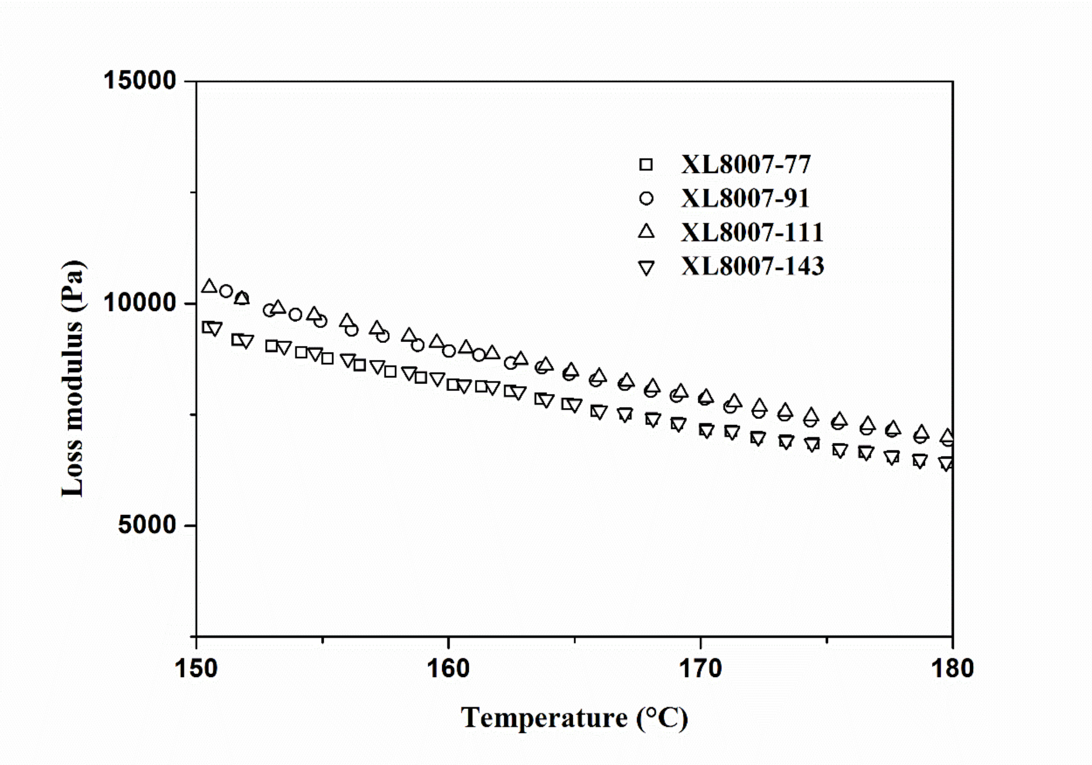

Figure S2. Effects of loss modulus by the peroxide mixture content for HDPE 8007.

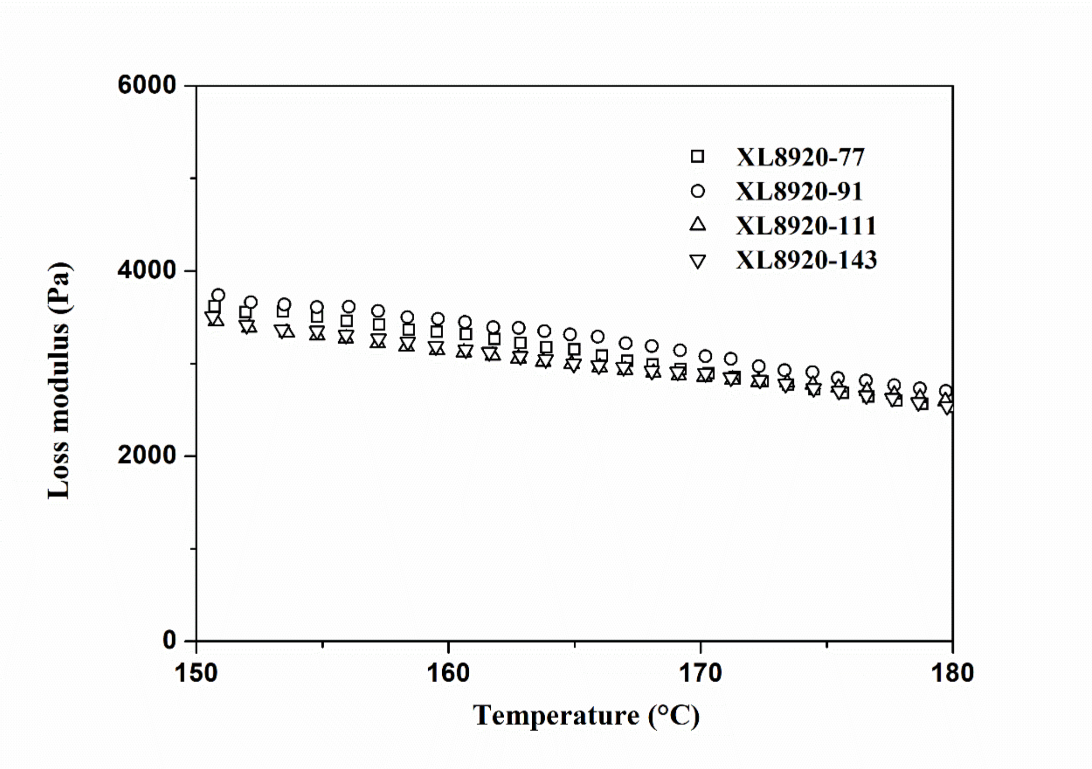

Figure S3. Effects of loss modulus by the peroxide mixture content for HDPE 8920.

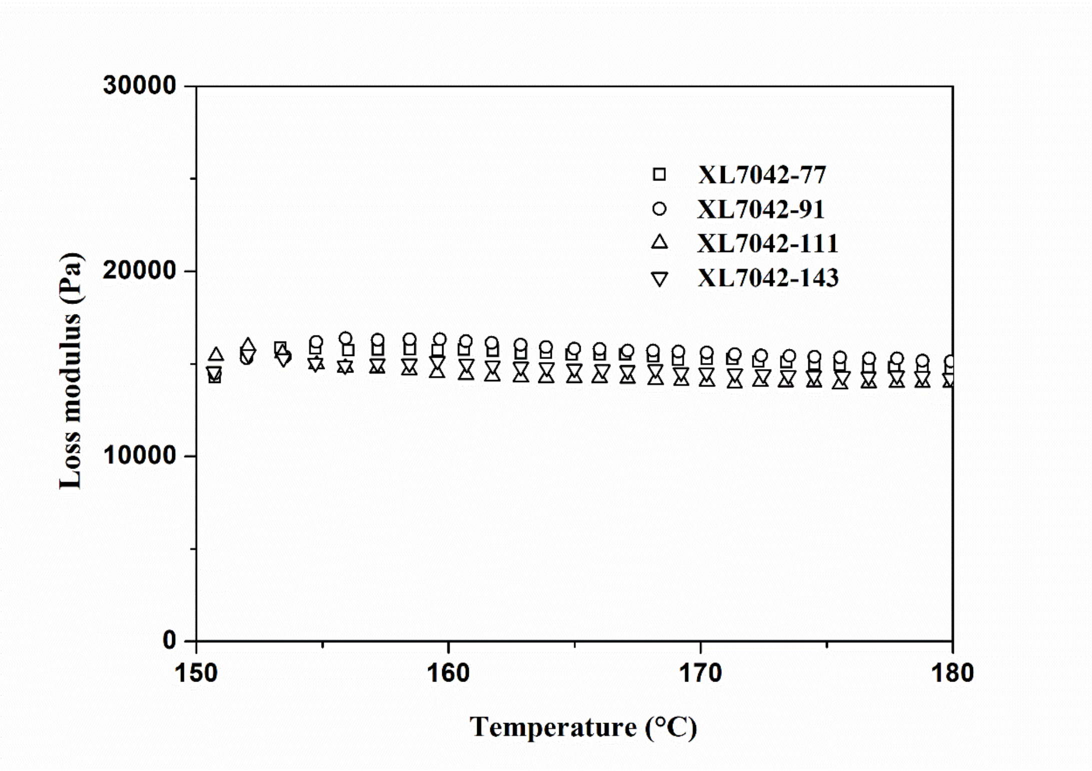

Figure S4. Effects of loss modulus by the peroxide mixture content for LLDPE 7042.

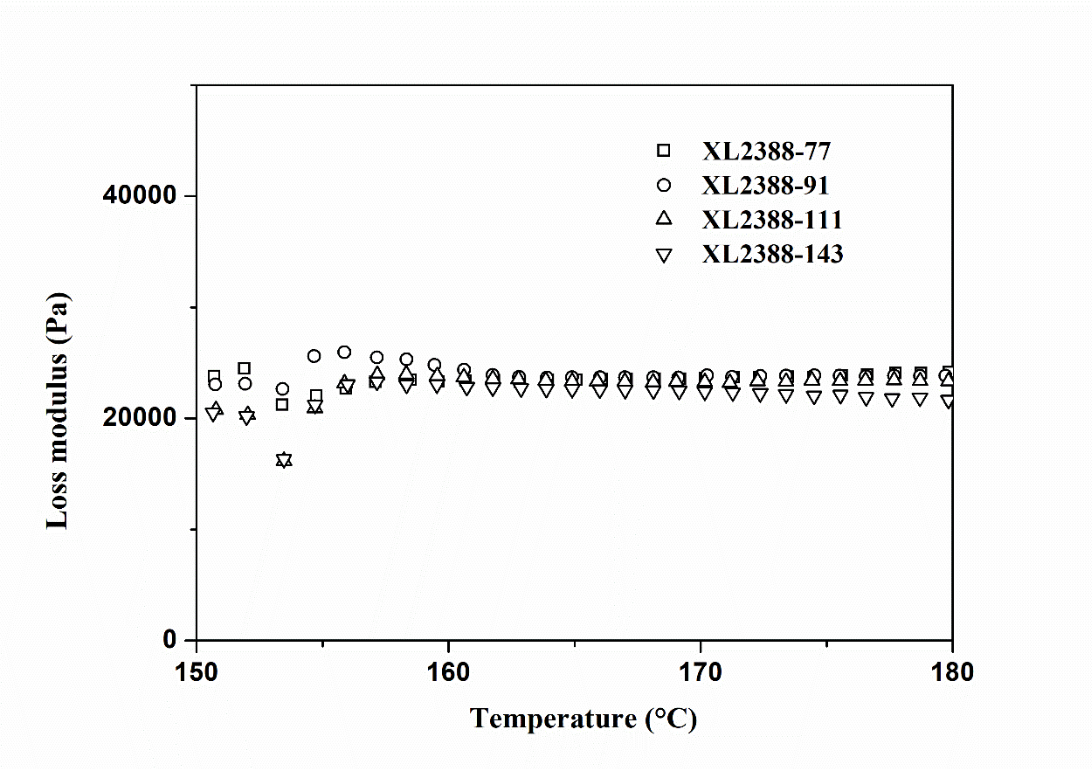

Figure S5. Effects of loss modulus by the peroxide mixture content for MDPE 2388.
